# Improving Iturin A Production of *Bacillus amyloliquefaciens* by Genome Shuffling and Its Inhibition Against *Saccharomyces cerevisiae* in Orange Juice

**DOI:** 10.3389/fmicb.2018.02683

**Published:** 2018-11-08

**Authors:** Juran Shi, Xiaoyu Zhu, Yingjian Lu, Haizhen Zhao, Fengxia Lu, Zhaoxin Lu

**Affiliations:** ^1^College of Food Science and Technology, Nanjing Agricultural University, Nanjing, China; ^2^Department of Nutrition and Food Science, University of Maryland, College Park, MD, United States

**Keywords:** genome shuffling, iturin, preservation, orange juice, *Saccharomyces cerevisiae*, *Bacillus amyloliquefaciens*

## Abstract

Genome shuffling is an effective method for the rapid improvement of the production of secondary metabolites. This study used the principle of gene shuffling to enhance the yield of iturin A produced by *Bacillus amyloliquefaciens* LZ-5. Improvements in lipopeptide yield were evident among four strains subjected to recursive protoplast fusion. The four strains were obtained through mutagenesis processes: nitrosoguanidine, ultraviolet irradiation, and atmospheric and room temperature plasma. A high yield strain with 179.22 mg/l of iturin A was obtained after two rounds of genome shuffling, which was a 2.03-fold increase compared with the wild strain. To evaluate the efficacy of iturin A for control of spoilage yeast in food, the anti-yeast efficacy of iturin A was evaluated in orange juice incubated with *Saccharomyces cerevisiae*. The juice treated with 0.76 mg/ml iturin A showed a significant (*p* < 0.05) control on yeast population during the storage, similar to that of the 0.30 mg/ml natamycin. In addition, iturin A showed a tiny effect on chemical-physical characteristics of orange juice. Our results provide a basis for the application of antimicrobial lipopeptide in juice products.

## Introduction

*Bacillus* strains can produce many kinds of secondary metabolites including antimicrobial lipopeptides synthesized by a complex multifunctional enzyme system. They are also reported to have a genomic basis for the biosynthesis of polyketide or nonribosomal peptide derivatives due to the presence of polyketide synthases (PKSs) and nonribosomal peptide-synthetase (NRPS). Iturin A, which is a cyclo-lipopeptide containing seven residues of α-amino acids (L-Asn-D-Tyr-D-Asn-L-Gln-L-Pro-D-Asn-L-Ser-) and one residue of a β-amino acid, is an essential ingredient of the antimicrobial substance. Because of its strong antifungal activity against an extensive range of plant pathogens, iturin A is applied the most widely in biological control of plant diseases (Leclere et al., 2005). Moreover, antimicrobial lipopeptides are also used in the cosmetic industries (Kanlayavattanakul and Lourith, 2010), food (Bie et al., 2005). And they can enhance oil recovery (Schaller et al., 2004) and improve the bioremediation of oil-contaminated sites (Mulligan et al., 2001). Iturin A is produced mainly by *Bacillus amyloliquefaciens* and *B. subtilis*, but its application is limited by low production.

During the last decade, researchers have made good efforts in improving the production of iturin A. The optimization of medium and culture conditions is in general use, and the screening of wild strains (Cho et al., 2009) with high yield is another critical method. But these rational methods and traditional techniques are inefficient (Grau et al., 2000). As the reach of shuffling technology from DNA fragments expands to the whole genome, genome shuffling is regarded as an alternative means to wild strain improvement. It could accelerate evolution without the need of sequence information. Genome shuffling is an efficient method that uses successive protoplasts fusion to recombine useful gene segment of parental strains into one single cell showing the desired phenotype (Biot-Pelletier and Martin, 2014). This approach was first reported by Zhang et al. (2002), who carried out two rounds of shuffling of *Streptomyces fradiae*, acquiring a sixfold increase in the production of tylosin in comparison with the parental strain. Additionally, genome shuffling is widely applied in sugar alcohol production with the improvement of yeast *Pichia anomala* (Zhang et al., 2015), the ethanol tolerance increase in *Saccharomyces cerevisiae* (Snoek et al., 2015), the butanol production enhancement of *Clostridium acetobutylicum* (Li et al., 2016),and the improvement of L-lactic acid production from the fusant of *Lactobacillus delbrueckii* and *B. amyloliquefaciens* (John et al., 2008).

The inherent acidity of fruit juices provides a strong defense to most bacterial species. However, several outbreaks of yeast infections have occurred recently. It is becoming a detrimental issue to fruit products as yeasts have tolerance to acidity (Aznar et al., 2015). According to the report, the spoilage of fresh pineapple juice is generally attributed to *Pichia guilliermondii*, *P. fermentans*, *P. membranifaciens*, *Hanseniaspora uvarum*, *Candida stellata*, *Rhodotorula spp*. (Bevilacqua et al., 2015). Moreover, *S. cerevisiae* and *Schizosaccharomyces pombe* can spoil most kinds of fruit juices and soft drinks (Malcolm, 2006). And many studies have taken *S. cerevisiae* as the primary focus in the microorganism control of orange juice (Loredo et al., 2015; Samani et al., 2015). The existence of this contamination will cause potential health hazards and serious economic losses. In general, the control of spoilage yeasts in food and beverage manufacturing is managed mainly by using some commercial food-grade chemical additives (Labbani et al., 2015) or thermal treatments. However, these measures may have harmful effects on human health or make changes in bioactive compounds present as well as any organoleptic changes. In the past decades, there is a growing popularity in finding alternatives, including vanillin, benzaldehyde, ferulic acid (Bevilacqua et al., 2015), and mentha oil (Tyagi and Malik, 2010). These alternatives are used to avoid the defect of conventional methods. Lastly, new natural anti-yeast compounds are still promoted for extending the shelf life of fruit juice and for avoiding microorganism infections.

The molecular structure of antimicrobial lipopeptide is of great interest because of their biological and physicochemical properties, which can be applicable in food, oil, and pharmaceutical industries (Meena and Kanwar, 2015). Up to now, antimicrobial lipopeptide is mostly considered as a biosurfactant for applications in food industry (Nitschke and Costa, 2007). It is used as emulsifiers in the processing of raw materials; in order to maintain texture, stability, and volume especially in baking. Furthermore, it helps with the emulsification of fat in order to control the aggregation of fat globules (Mandal et al., 2013). The existence of lipopeptides in fermented food products (Cho et al., 2009) was also regarded for their applications in food. On the contrary, there is little study on its antimicrobial activity in fruit juice, where there is an acid condition for lipopeptide stability. According to our knowledge, some kind of iturin exerts the activity of anti-yeast, including iturin A, bacillomycin F (Mhammedi et al., 1982), mycosubtilin (Leclere et al., 2005), etc. In that respect, there is a potential application in the control of yeast spoilage in food. Notably, iturin is natural and would replace the use of chemical preservatives. In the present study, we tried to use the mean of genome shuffling to increase the yield of iturin A from *B. amyloliquefaciens*. Moreover, we intend to investigate its potential in the preservation of orange juice against *S. cerevisiae* and evaluate its effect on the chemical-physical properties of juice.

## Materials and Methods

### Strains and Culture Conditions

The wild strain, *B. amyloliquefaciens* LZ-5 was isolated from Chinese honey. Three mutagenesis methods, nitrosoguanidine (NTG),ultraviolet irradiation (UV),and atmospheric and room temperature plasma (ARTP) were used to acquire four mutants. The average antimicrobial lipopeptide production of these mutants was 23.1% higher than the wild strain and they were used as the initial strains. The mutants were maintained in the standard potato dextrose agar (PDA) media at 37°C, and the inoculums were prepared in potato dextrose broth (PDB) media shaking at 180 rpm at 37°C for 12 h. The modified landy medium (glucose 42 g/l, L-sodium glutamate 14 g/l, MgSO_4_ 0.5 g/l, KCl 0.5 g/l, KH_2_PO_4_ 1.0 g/l, FeSO_4_ 0.15 mg/l, MnSO_4_ 5.0 mg/l, CuSO_4_ 0.16 mg/l) was used as fermentation medium. SMM (sucrose 171.14 g/l, MgCl_2_⋅6H_2_O 4.07 g/l, and maleic acid 2.32 g/l) was taken as a stabilizer. The protoplast was maintained on hypertonic regeneration medium (RM) medium (PDA with 0.6 mol/l NaCl) for cell regeneration. Lysozyme was purchased from Sigma and dissolved in PSB, sterilized by filtration through a 0.22 μm membrane filter. Polyethylene glycol (PEG) 6000 (40%) was prepared in SMM. *S. Cerevisiae* (AS2.114), preserved in PDB media with 20% (v/v) glycerol at -20°C, was the indicator to evaluate the effect of iturin A. Before use, the yeast strain was cultured in PDB media at 28°C for 24 h at 180 rpm. In broth microdilution assay, *S. cerevisiae* was cultured in YPD (yeast extract 10 g/l, peptone 20 g/l, glucose 20 g/l) media. All other chemicals used in this work were of analytical grade.

### Procedure of Genome Shuffling

The parent library was constructed by UV, NTG, and ARTP mutagenesis methods and four strains named U-36, N-22, A-8, and A-61 were collected in the previous work as the initial strains. The initial strains were cultured for 8 h at 37°C in 50 ml PDB medium. When the optical density at 600 nm (OD_600_) reached 1.6∼1.8, cells were harvested by centrifugation, washed twice with sterile double-distilled water and suspended in SMM in a final concentration of 10^7^ cells/ml. The cell suspensions were incubated in PSB containing 0.2 mg/ml lysozyme in a water bath at 37°C for 10 min. After the enzymatic digestion of the cytoderm, cells were washed twice and re-suspended in PSB. The formation of protoplasts could be judged by light microscopy.

The equal number of protoplasts from different populations were mixed and divided into two fractions for inactivity. One fraction was placed under a preheated 30 W UV lamp at a vertical distance of 15 cm and irradiated for 60 min. The other was put in a water bath at 100°C for 30 min. All of the inactivated protoplasts were mixed in the same ratio, washed twice, centrifuged and re-suspended in 0.5 ml SMM. After the addition of 4.5 ml 40% (v/v) PEG 6000, the suspension was shaken gently at room temperature for 5 min to allow protoplasts fusion. After adding 5 ml PSB to terminate the reaction, the fused protoplasts were washed, centrifuged and re-suspended in 1 ml PSB. The serial dilutions of the suspension were spread on RM plates and incubated for 36 h at 37°C. The antimicrobial lipopeptide productivity was determined by the agar-diffusion method employing *S. cerevisiae* as an indicating strain. After preliminary screening, the production of iturin A in selected strains was analyzed by HPLC (AGILENT 1100 series). Three strains with highest antimicrobial lipopeptide production were obtained as start strains for the next fusion. Two successive rounds of protoplast fusion were then carried out.

### Determination of Iturin A

The bioactive substance was isolated by acid precipitation. Activated culture of *B. amyloliquefaciens LZ-5* was incubated (5%, v/v) in modified landy medium, and shaken at 180 rpm at 30°C for 72 h. And then the fermentation broth was centrifuged at 10,000 *g*, 4°C for 20 min. The supernatant collected was adjusted to pH 2 using 6 N HCl, followed by centrifugation at 8000 *g* for 10 min. The supernatant was discarded and the precipitated lipopeptides were extracted for three times by anhydrous ethanol. To study the bioactive substance purified from fermentation broth, Iturin A was identified and measured by reversed-phase high-performance liquid chromatography (HPLC; C18 column, ODS 4.6 mm × 250 mm, AGILENT 1100 series) with UV detectors and HPLC-MS/MS (Thermo Electron Corporation, San Jose, CA, United States). The eluent was methyl cyanides at a flow rate of 0.6 ml/min. The injection volume of the sample was 20 μL. For its application in the orange juice, the bioactive substance was decolorized using 0.5% (w/v) activated carbon and gently shaken for 4 h, and then it was concentrated and freeze-dried. Then anti-yeast test of Iturin A in Orange Juice was carried out as following.

### Treatment of Orange Juice With Iturin A

The commercial orange juice used in this study was purchased from a local store in Nanjing, Jiangsu province, China. 50 ml of orange juice was placed in sterilized glass vials. The suspension of *S. cerevisiae* was added to orange juice resulting in final concentration of 10^3^ cfu/ml. The iturin A was dissolved in 0.5 ml ethanol and then mixed with orange juice resulting in three final concentrations of 0.13, 0.38, and 0.76 mg/ml, respectively. Natamycin was added into the orange juice at the final concentration of 0.3 mg/ml. Six treatments, including a blank control (juice plus sterile water), negative control (juice plus 10^3^ cfu/ml *S. cerevisiae* and sterile water), Natamycin (juice plus 10^3^ cfu/ml *S. cerevisiae* and 0.3 mg/ml natamycin), C1 (juice plus 10^3^ cfu/ml *S. cerevisiae* and 0.13 mg/ml iturin A), C2 (juice plus 10^3^ cfu/ml *S. cerevisiae* and 0.38 mg/ml iturin A), and C3 (juice plus 0.76 mg/ml iturin A and 10^3^ cfu/ml *S. cerevisiae*), were set in this study. The prepared orange juice was stored at 25°C for 10 day and the quality of samples were detected on 0, 2nd, 4th, 6th, 8th, and 10th day, respectively.

### Effect of Iturin A on Yeast Growth

0.1 ml aliquot of each sample was serially diluted and plated on PDA. The plates were incubated for 24 h at 28°C and the colonies were counted. The efficacy of different concentration of iturin A and natamycin was indicated by the variation of the inoculated yeast strains with storage time, which is expressed in log cfu/ml (Tyagi et al., 2013).

### Physical-Chemical Characteristics of the Stored Orange Juice

A set of the orange juice samples were incubated with *S. cerevisiae* of 10^3^ cfu/ml and the total acidity,vitamin C, total soluble solid and browning index were measured. The total acidity was assessed by direct titration referring to the modified method described by Peter et al. (2012). 5 ml of juice was diluted with ultrapure water up to 100 ml. Then,a volume of25 ml solution was placed in an erlenmeyer flask and two drops of phenolphthalein solution 1% were added. The mixture was titrated with 0.1 N NaOH solution, until the pink color of the solution persisted for at least 1 min. Titratable acidity was expressed as grams malic acid per 100 ml juice.

The vitamin C was measured using the 2,6-dichloroindophenol titrimetric method according to Masamba and Nguyen (2008). A quantity of 5 ml juice was diluted with 2% solution of oxalic acid up to 100 ml and the mixture was centrifuged at 4,000 rpm for 5 min. 10 ml of supernatant was titrated with the standard solution 2,6-dichloroindophenol (0.1%) until the pink color of the solution persisted for at least 1 min. The vitamin C content was expressed as milligrams ascorbic acid per 100 ml juice.The total soluble solids (TSS) were measured using a digital refractometer (WZS-1, Shanghai, China) and the TSS values were reported as ^o^Brix. The browning index was also determined using the previously reported method of Meydav et al. (1977).

### Statistical Analysis

All of the experiments were repeated three times and each repetition was conducted in triplicate. For physico-chemical properties analysis, the results obtained were submitted to one way ANOVA (*p* < 0.05) using SPSS version 20. In the figures, data, and error bars represent arithmetic mean values and standard deviation, respectively.

## Results

### Genome Shuffling of *B. amyloliquefaciens* for Improving Iturin A Production

**FIGURE 1:**
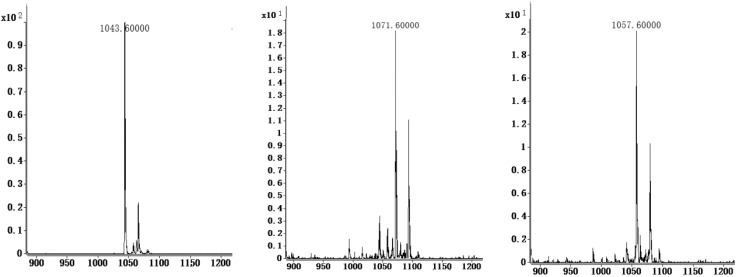
The LC-MS spectrum of iturin A homolog from *Bacillus amyloliquefaciens LZ-5.*

*B. amyloliquefaciens* LZ-5 isolated from Chinese honey was found strong anti-yeast activity. Then the anti-yeast compounds were separated and purified, and three substances were elucidated by HPLC-MS/MS. The positive ion mass spectrum analysis showed that [M+H]^+^ ion peaks were 1043.6, 1057.6, and 1071.6, respectively. And they were identified as to be Iturin A homolog, iturin A2, iturin A3, and iturin A4, with molecular weight of 1042.6, 1056.6, and 1070.6 Da (Figure 1).

**FIGURE 2:**
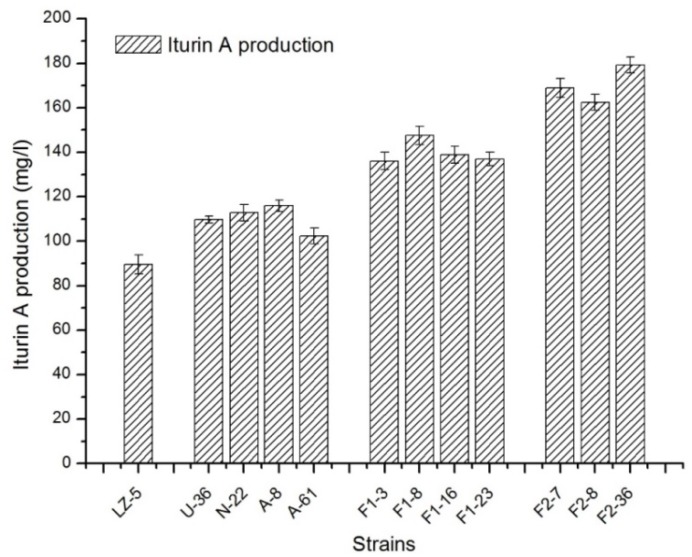
The production of iturin A in different strains of *B. amyloliquefaciens* in landy medium. LZ-5, wild-type of *B. amyloliquefaciens*; U, N, A, the mutant strains of UV, NTG, and ARTP, respectively; F1, strains from the first round of genome shuffling; F2, strains from the second round of genome shuffling.

To enhance the yield of iturin A, genome shuffling method was applied and two successive rounds of genome shuffling were carried out in this study. The four mutants of NTG, UV, and ARTP collected previously named U-36,N-22,A-8, A-61 were subjected to a first round of pool-wise recursive protoplast fusion. As shown in Figure 2, the production of iturin A was improved steadily after each turn of gene fusion. During the screening of the first generation, 325 colonies were picked out from the PDA medium with 0.6 mol/l sodium chloride for the fermentation test. And four isolates F1-3, F1-8, F1-16, and F1-23 were selected for the detection of iturin A concentration by HPLC. Their iturin A production was in the range of 136.05∼147.42 mg/l, which was increased 51.9–64.68% as compared with the wild strains. And the four strains were used for the next shuffling. After the second shuffling, three isolates, F2-7, F2-8, and F2-36, with significantly increased iturin A production were obtained. The strain F2-36 exhibited 179.22 mg/l iturin A yield, which was a 2.03-fold increases compared to *B. amyloliquefaciens LZ-5*.

### Effect of Iturin A on Growth of Yeast in Orange Juice

**FIGURE 3:**
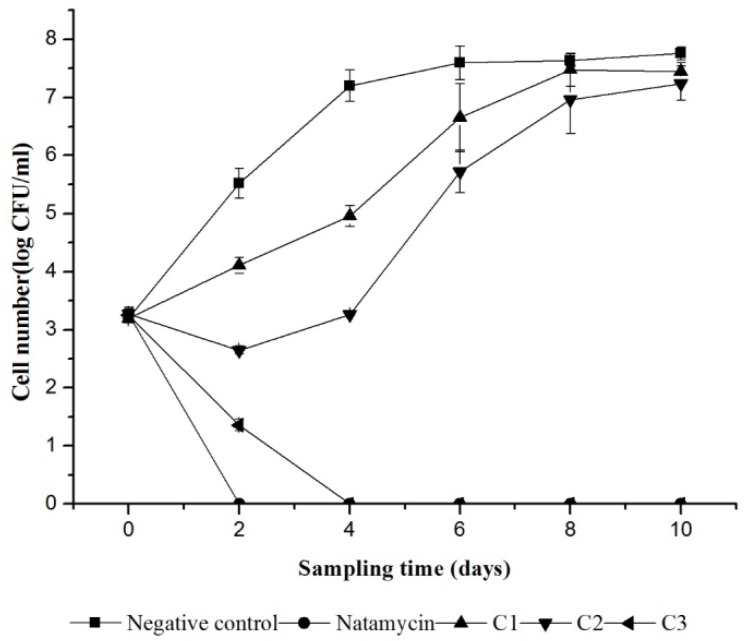
Variation in viability of *S. cerevisiae* in orange juice during storage at 25°C. Natamycin treatment at 0.3 mg/ml; Negative control treated with sterile water. Iturin A treatment at 0.13 mg/ml (C1), 0.38 mg/ml (C2),and 0.76 mg/ml (C3).

In order to evaluate the inactivation on *S. cerevisiae* by iturin A and the preservation of orange juice, samples were inoculated with spoilage organisms. Figure 3 shows the effect of different treatments on *S. cerevisiae* survival during storage at 25°C. The initial count of yeast in different treatments ranged from 3.20 log cfu/ml to 3.28 log cfu/ml except with the blank control which contain no treatment. The application of iturin A had a significant (*p* < 0.05) effect on the population of yeast in orange juice compared to the water as a control, in which the count of yeast reached 7.60 log cfu/ml in 6 days. A complete inhibition was occurred in 4 days when the additive amount of iturin A was 0.76 mg/ml (C3). It is observed that yeast growth in the sample added with natamycin (0.3 mg/ml) and iturinA (0.76 mg/ml) was fully inhibited. However, the inhibition effect was decreased gradually as the concentration of iturin A decreases (Figure 3).

### Physico-Chemical Properties of the Stored Orange Juice

**FIGURE 4:**
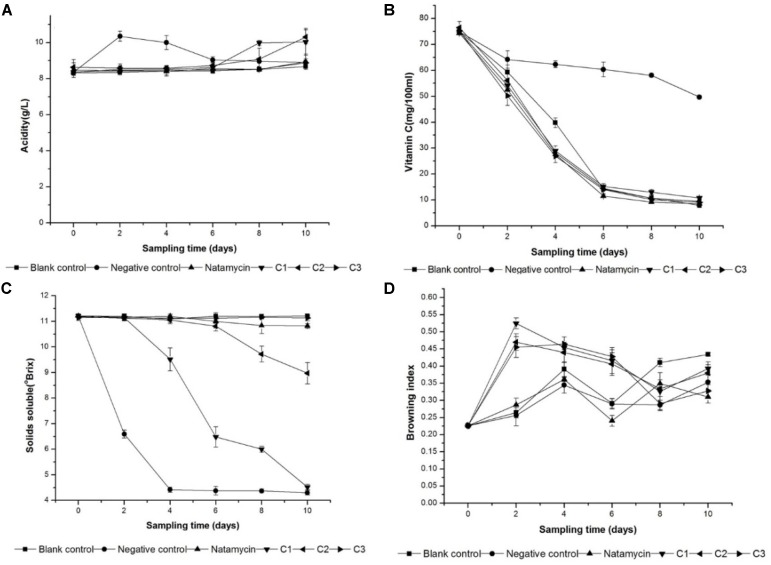
Changes in Vitamin C **(A)**,the total acidity **(B)**,the total soluble solid **(C)**, and browning index **(D)** of orange juice during storage at 25°C. Natamycin with the concentration at 0.3 mg/ml. Negative control and blank control treated with sterile water. iturin A treatment at 0.13 mg/ml (C1), 0.38 mg/ml (C2), and 0.76 mg/ml (C3).

The physico-chemical properties can be used to evaluate the freshness of orange juice. Total titratable acidity, vitamin C, total soluble solids (TSS) and the browning index were monitored during the storage. As Figure 4A showed, the total titratable acidity had no significant (*p* < 0.05) difference in blank control, natamycin, and C3 after 10-days storage. There were varying degrees of increases between the other three groups because of acidic compound production by microbial fermentation. For example, the negative control incubated with yeast had a significant increase (*p* < 0.05) at the 2nd day, and the yeast incubated in C1 and C2 started to reproduce resulting in a sudden increase (*p* < 0.05) of the total titratable acidity in the last 2 days. It can indicated that the existence of natamycin and 0.76 mg/ml iturin A inhibit the fermentation progress.

The vitamin C content reduced gradually as days of storage increased. In all samples, the reduction was ranged from 50∼70% in different groups. The changes in vitamin C concentration during orange juice storage were analyzed and are depicted in Figure 4B. The rate of reduction of the sample treated with iturin A and natamycin was keep in pace with the blank control. This can indicate that there is no effect on the concentration of vitamin C in orange juice in the presence of iturin A. However, in the negative control, the reduction in vitamin C content was only 30% during the storage time, which was significantly (*p* < 0.05) lower than the other groups.

Figure 4C illustrates the variation of the total soluble solids (°Brix) of the orange juice samples during storage in different treatments. The total soluble solids (°Brix) is the reflection of sugar content to a large extent. And there was no significantly difference in groups with no microbial fermentation including the blank control, natamycin, and C3. In the sample treated with sterile water, the total soluble solids (°Brix) was reduced (*p* < 0.05) to a lowest level at the 4th day, and that of the other group treated with iturinA at a concentration of 0.38 and 0.13 mg/ml was also decreased (*p* < 0.05) to different degree.

When the browning index was analyzed after 10 days of storage, there was an increase (*p* < 0.05) in this index at the 2-days storage in the group treated with iturin A (Figure 4D). This change had nothing to do with the concentration of iturin A. In the end of the storage, there was no statistically significant difference (*p* < 0.05) observed between the samples. The presence of iturin A probably increased the rate of the browning reactions compared to the natamycin.

## Discussion

Genome shuffling is an efficient way for the evolution of new strains and the improvement of secondary metabolites. This strategy has been used in an attempt to enhance the production of fengycin and surfactin (Zhao et al., 2012, 2016). In this study, we utilized this advantage of genome shuffling to enhance the yield of iturin A in *B. amyloliquefaciens* with an increase of 2.03-fold. Up to now, there is no relative report about application of genome shuffling in iturin A improvement. The traditional optimization strategy, often requires a considerable amount of work and time, and constantly fails to yield optimized conditions because it involves in more than one variable. Medium optimization of iturin A production has been carried out in solid-state fermentation using response surface methodology by Mizumoto and Shoda (2007), there was an 2.07-fold increase compared to the initial medium, but this satisfactory result was obtained through a great number of experiments and required a higher cost. However, genome shuffling amplifies genetic diversity by homologous recombination using protoplast fusion within the selected mutant population (Hida et al., 2007). By combining the three different kinds of mutagenesis which can effectively create more genetic variety (Zhao et al., 2012; Fang et al., 2013), the parent library with high productivity was established by using mutation-induced technique with UV, NTG, and ARTP in the work. Additionally, the successive improvement of the population was obtained by successive genome shuffling according to Patnaik et al. (2002), etc. Two successive rounds of gene shuffling were carried out in total with the efficiently screening strategies of agar diffusion. A new isolate (F2-36) exhibited significantly improved productivity of iturin A by 2.03-fold. Therefore, genome shuffling, based on the protoplast fusion involved in the whole genome, is more convenient and simple in the improvement of iturin A yield.

Considering the anti-yeast activity of iturin A, iturin A, obtained by acid precipitation from fermentation broth, was tested against *S. cerevisiae* to evaluate the inoculated orange juice. *S. cerevisiae* are major spoiled strains (Malcolm, 2006) in juice products, and the results of *in vitro* tests indicated that iturin A completely suppressed the growth of *S. cerevisiae* incubated in juice stored at 25°C with a concentration of 0.76 mg/ml at the 4th day, which was similar to the efficiency treated with 0.3 mg/ml natamycin. To our best knowledge, limited report was found about this natural compound tested *in vivo* as a preservative to reduce yeast contamination. But for seeking for an alternative of chemical additives, some other kinds of natural product have been studied. Among them, lemon grass oil at 1.13 mg/ml exerted complete growth inhibition of *S. cerevisiae* in mixed fruit juice (Tyagi et al., 2014), and the MFC of *Eucalyptus globulus* oil against *S. cerevisiae* was 2.25 mg/ml (Tyagi and Malik, 2011), etc. These natural compounds have different levels of inhibition effect on the yeast cell, and the extent of inhibition increases with increasing concentration of all natural compounds. Whereas iturin A from a microorganism is easier to achieve an efficient production than from the extraction of plants, so its application in food is more potential.

The physical-chemical parameters are the most representative to characterize the orange juice. In this study, the total acidity, vitamin C, the total soluble solid and the browning index were monitored during the storage of the orange juice. The total acidity and the total soluble solid nearly had no change in the 10-days storage at 25°C after C3 treated with iturin A. This result was in accordance with the previous study (de Oliveira Junior et al., 2015), where there was no changes in total acidity in juice without fermentation progress. Yeast growth can remove a small proportion of the sugars in a fruit juice (Malcolm, 2006), which is closely related to the amount of the total soluble solid. Therefore, subtle changes have been observed in juice added with iturin A in comparison to the negative control. Vitamin C loss follows a linear equation by applying a regression analysis with increasing storage time on account of oxidative enzyme reaction (Lee and Coates, 1999). This gives a suitable explanation to our result that the content of vitamin C was decreased in all samples during 10-days storage. Regarding to the browning index, there is hardly any significant difference among these groups.

## Conclusion

This study demonstrates that the gene shuffling is an effective method to accelerate the evolution of strains by improving the iturin A production in *B. amyloliquefaciens*. Furthermore, we introduced the antimicrobial lipopeptide in the preservation of juice product for the first time. The experimental evidence shows that all concentrations of iturin A have an effect against the *S. cerevisiae* incubated in orange juice, without hardly changing the physical and chemical properties of the sample. The iturin A has the potential as an additive to control the yeast contamination in juice products. Further research should be pursued in order to investigate the impact of iturin A on human and animal cell lines.

## Author Contributions

ZL designed the research. JS, XZ, and YL performed the research. JS, XZ, YL, HZ, and FL analyzed the data. ZL and JS wrote the paper.

## Conflict of Interest Statement

The authors declare that the research was conducted in the absence of any commercial or financial relationships that could be construed as a potential conflict of interest.
